# Early Staged Fixation of an Open Pilon Fracture After a Motor Vehicle Collision

**DOI:** 10.7759/cureus.119558

**Published:** 2026-11-12

**Authors:** Ryan Magee, Levi Streit, Laurel Marsh, Jason Deck

**Affiliations:** 1 Department of Family and Sports Medicine, University of Oklahoma School of Community Medicine, Tulsa, USA

**Keywords:** diabetes mellitus, external fixation, fibular fracture, limited internal fixation, open pilon fracture, open reduction and internal fixation, staged fixation, tibial plafond fracture

## Abstract

Pilon fractures challenge reconstruction because the articular injury occurs within a thin, vulnerable soft tissue envelope, and the optimal timing of definitive fixation after an open injury remains uncertain. We describe a 56-year-old man with type 2 diabetes mellitus who sustained an open right pilon fracture with an associated distal fibular fracture in a restrained head-on motor vehicle collision. Intravenous antibiotics were given shortly after arrival, and the fracture was closed-reduced and temporarily immobilized in the trauma bay. The next day, he underwent irrigation and debridement, percutaneous lag screw fixation of a posterior component, and ankle-spanning external fixation. Four days later, definitive open reduction and internal fixation of the tibia was performed through anterolateral and posteromedial approaches, followed by frame removal; the fibula was managed non-operatively. At three weeks, the incisions showed no signs of infection, the traumatic wound had a dry eschar, and peri-incisional sensation was decreased. At eight weeks, there was still no clinical evidence of infection, and radiographs showed intact hardware and interval callus with some shortening and a slight extension deformity. This case illustrates individualized early conversion within a staged strategy in a patient at increased risk of complications, and it raises the question of fibular fixation for maintaining length. Eight-week follow-up cannot establish union, function, or an optimal interval; timing should remain guided by the wound, soft tissues, fracture pattern, and patient condition.

## Introduction

Pilon fractures affect the distal tibial plafond and commonly result from axial loading during motor vehicle collisions or falls from height. The fracture and its thin soft tissue envelope must be considered together because swelling, open wounds, contamination, and fracture morphology influence both surgical approach and timing [1].

For high-energy injuries or compromised soft tissues, staged treatment with temporary ankle-spanning external fixation followed by open reduction and internal fixation (ORIF) is widely used [1,2]. Open pilon fractures remain heterogeneous, and the interval to definitive fixation varies according to wound severity, swelling, contamination, planned incisions, and the need for soft tissue coverage. A systematic review of 484 open pilon fractures emphasized both the substantial complication burden and the limited comparative evidence available for definitive fixation strategies [3].

This report describes an open pilon fracture in a patient with diabetes, managed with debridement, limited internal fixation, and temporary external fixation, followed by definitive ORIF four days later. The report focuses on the staged sequence, the early wound outcome, and early radiographic findings; it does not propose a universal four-day conversion interval.

## Case presentation

A 56-year-old man presented to the emergency department after a restrained head-on motor vehicle collision with an open injury of the right ankle. His outpatient medications included metformin, pioglitazone, and dulaglutide for type 2 diabetes mellitus, as well as amlodipine, losartan, hydrochlorothiazide, and simvastatin. He reported never using tobacco or alcohol. Examination documented an obvious deformity, right ankle tenderness, and a small poke hole wound on the medial aspect of the ankle. Intravenous ampicillin-sulbactam was administered within 30 minutes of arrival. The fracture was closed-reduced and immobilized in the trauma bay, and postreduction radiographs and non-contrast computed tomography of the right ankle were obtained. The reduction maneuver and initial immobilization device were not specified in the available record. Initial radiographs demonstrated a displaced, comminuted distal tibial plafond injury with an associated displaced distal fibular fracture (Figure 1). Initial documentation described a Type I or II open displaced pilon fracture; a subsequent orthopedic progress note documented the postoperative diagnosis as a Type I open right pilon variant, although wound size alone does not establish the Gustilo-Anderson grade; high-energy trauma can warrant Type IIIA classification regardless of wound size [4]. An AO/Orthopaedic Trauma Association subtype was not documented in the available clinical record.

**Figure 1:**
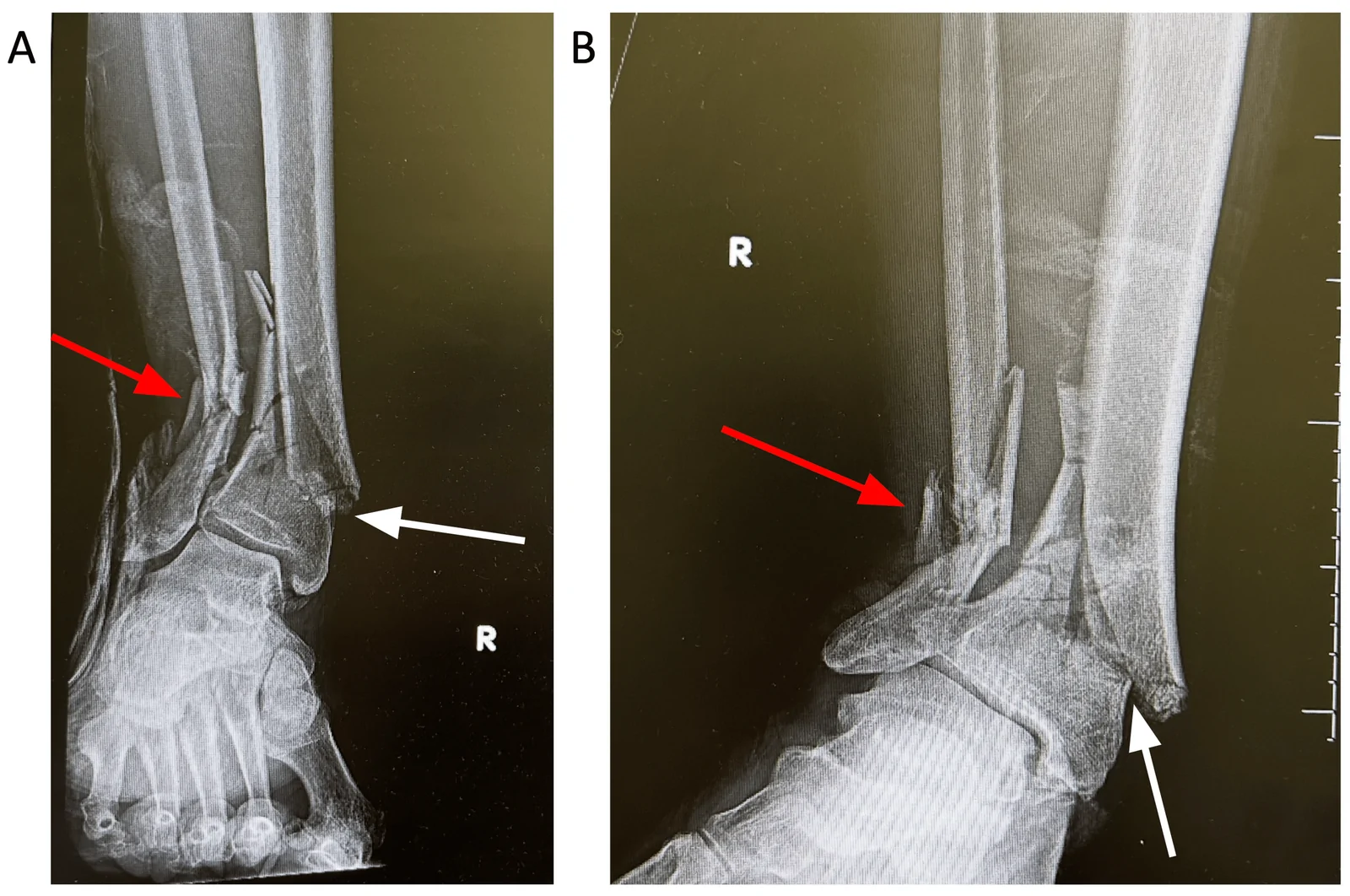
Initial anteroposterior and lateral radiographs of the right ankle The anteroposterior (A) and lateral (B) projections demonstrate a displaced, comminuted distal tibial plafond injury (white arrows) and an associated displaced distal fibular fracture (red arrows). The open fracture classification is based on the clinical documentation rather than the radiographs.

The following day, irrigation and debridement were performed. The anteromedial wound was extended to approximately 1 × 1 cm, followed by debridement from superficial to deep and irrigation; this dimension describes the wound extension, not the extent of tissue debrided. A posterior fracture component was reduced, and a 2.7-mm screw was inserted percutaneously using lag-by-technique fixation. After screw placement, an ankle-spanning external fixator was applied under fluoroscopic guidance. Bicortical 5-mm Schanz pins were placed through the anteromedial tibia, and a centrally threaded 5-mm transcalcaneal pin was placed in a safe zone. The frame was connected and adjusted to restore length and coronal alignment; an additional off-plane tibial pin was added for rotational stability.

Four days after the index operation, definitive ORIF of the tibia was performed under general anesthesia with a thigh tourniquet. Through an approximately 5-cm anterolateral incision, the distal tibia was exposed, the previously placed 2.7-mm screw was removed, and a 14-hole stainless steel anterolateral distal tibial plate (EVOS Small Fragment Plating System; Smith & Nephew, Memphis, Tennessee, United States) was positioned under fluoroscopy and held provisionally with Kirschner wires. After screw removal and provisional plate placement, the posterior malleolar component remained displaced and extended, so a posteromedial approach was used. The posterior tibialis tendon sheath was opened, the tendon was mobilized posteriorly, and the fracture was reduced with a Weber clamp under fluoroscopic guidance. Distal fixation included 2.7- and 3.5-mm cortical screws, with the 2.7-mm screws placed using lag-by technique; 3.5-mm cortical screws were placed proximally in a balanced fixation pattern. After final fixation and confirmation of length, alignment, and rotation, the external fixator was removed. The fibular fracture was not fixed. Tourniquet time was 121 minutes, estimated blood loss was 25 mL, and no intraoperative complications were recorded. A well-padded posterior short leg splint was applied after fixation (Figure 2).

**Figure 2:**
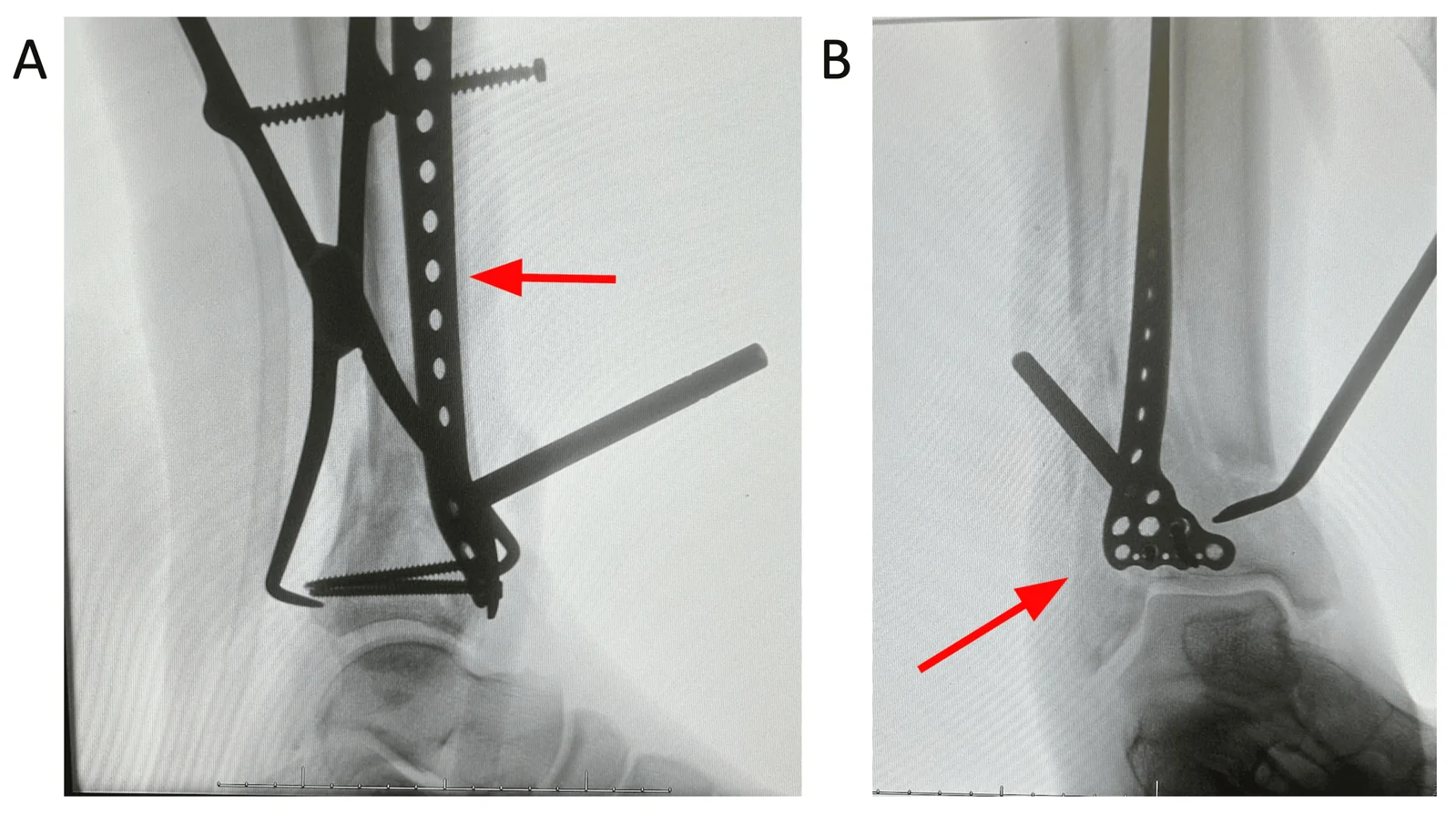
Intraoperative fluoroscopy during definitive fixation Lateral (A) and anteroposterior (B) fluoroscopic views show the anterolateral plate (red arrows) and provisional reduction instruments during reconstruction. The posteromedial exposure and posterior fragment reduction are described in the operative record.

Postoperative anteroposterior and lateral radiographs documented the definitive distal tibial plate and screw construct (Figure 3). These images demonstrate construct appearance but do not establish union or long-term maintenance of reduction.

**Figure 3:**
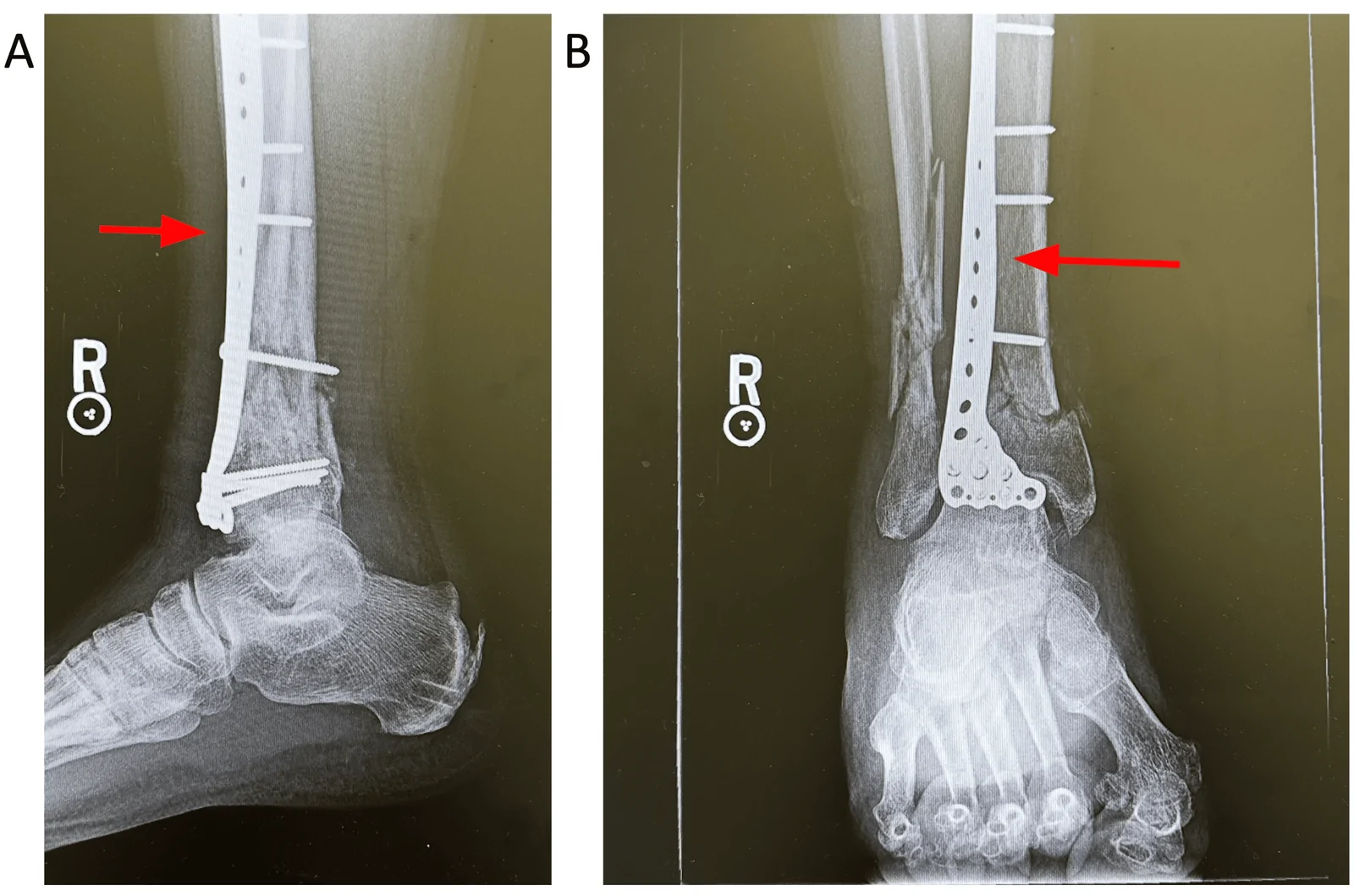
Postoperative anteroposterior and lateral radiographs of the right ankle The lateral (A) and anteroposterior (B) projections show the definitive distal tibial plate and screw construct (red arrows). No conclusion regarding union or long-term alignment can be drawn from these postoperative views alone.

The postoperative plan included non-weight-bearing for 6 to 12 weeks, postoperative antibiotics, venous thromboembolism prophylaxis, outpatient follow-up, and later transition to a removable boot. He was discharged home the day after definitive fixation with a referral for home health services.

At the three-week postoperative visit, the patient remained non-weight-bearing and was taking aspirin for venous thromboembolism prophylaxis. The surgical incisions were well approximated, with sutures still in place, and showed no signs of infection. A dry eschar remained over the anteromedial distal tibia at the open-fracture site. He could activate the extensor hallucis longus, flexor hallucis longus, and gastrocnemius-soleus complex. Sensation was decreased around the incision, but the extremity was otherwise documented as neurovascularly intact. Follow-up was planned in four weeks, with physical and occupational therapy to begin after the next appointment.

At eight weeks postoperatively, the patient reported adherence to non-weight-bearing, continued aspirin prophylaxis, and denied fevers, chills, or wound problems. Examination again documented no signs of infection at the incisions or the open-fracture site. Three radiographic views of the right ankle showed the anterolateral plate with intact screws and hardware and interval callus formation on the anteroposterior and lateral views; there also appeared to be some shortening at the fracture with a slight extension deformity. The fibular fracture appeared to be healing. Non-weight-bearing was continued for an additional four weeks, physical and occupational therapy were planned to begin after the next visit, and follow-up was arranged in six weeks with standing radiographs, including all hardware. No documentation beyond eight weeks was available for review.

The documented clinical course is summarized in Table 1.

**Table 1:** Clinical timeline ORIF, open reduction and internal fixation.

Time point	Documented findings and treatment	Plan or interpretation
Presentation	Restrained head-on motor vehicle collision; open right ankle deformity, medial poke hole wound, and tenderness; associated distal fibular fracture; intravenous ampicillin-sulbactam within 30 minutes of arrival	Closed reduction and immobilization in the trauma bay
Following day	Irrigation and debridement; percutaneous 2.7-mm lag screw fixation of a posterior component; ankle-spanning external fixation	Open wound care, provisional reduction, and temporary stabilization
Four days after index operation	Definitive tibial ORIF through anterolateral and posteromedial approaches followed by external fixator removal; fibula managed nonoperatively	Plate and screw reconstruction followed by a posterior short leg splint; discharged home the following day
Three weeks postoperatively	Non-weight-bearing; incisions approximated with sutures in place; dry eschar at the traumatic wound; no signs of infection; intact distal motor function; decreased peri-incisional sensation	Continued follow-up with rehabilitation planned after the next visit
Eight weeks postoperatively	Non-weight-bearing; no signs of infection; radiographs with intact hardware, interval callus, some shortening, and slight extension deformity; fibular fracture healing	Non-weight-bearing for four more weeks; therapy and standing radiographs planned at next visit

## Discussion

This case illustrates the combination of open fracture debridement, limited fixation of a posterior component, temporary ankle-spanning stabilization, and definitive ORIF four days later. This was an early staged conversion rather than acute definitive fixation: the index procedure addressed the wound and established temporary alignment while avoiding the broader exposures used during definitive reconstruction.

The timing of definitive fixation in pilon fractures is determined primarily by the condition of the soft tissue envelope rather than a fixed number of days. In the staged series by Sirkin et al., open pilon fractures underwent formal reconstruction a mean of 14 days after external fixation, with a range of 4 to 31 days [2]. Patterson and Cole reported a mean interval of 24 days for severe C3 fractures [5]. These cohorts involved different injury patterns and severities, but together they demonstrate that staged treatment encompasses a broad interval. A four-day conversion should therefore be understood as the lower boundary of a reported range, not a generally applicable target.

Limited fixation during the index procedure may be useful when a specific fragment can be reduced percutaneously without compromising later approaches. Hreha et al. compared traditional staged treatment with limited tibial reduction and fixation performed during initial external fixation in 113 patients [6]. Limited early fixation was associated with definitive ORIF 4.75 days sooner and a shorter definitive operation, without detected differences in infection, reduction quality, or nonunion. The retrospective design precludes causal conclusions. In this case, the initial screw was removed, and posterior reduction required further correction during definitive ORIF, so its specific contribution to staged management cannot be determined.

Open injuries require cautious comparison across studies. Olson et al. reported deep infection in 27% and nonunion in 22% among 161 complete articular open tibial plafond fractures, most of which were Gustilo-Anderson Type IIIA or IIIB; acute fixation of Type IIIA injuries was associated with more deep infection than delayed staged fixation [7]. The Type I diagnosis recorded in this case should not be used to assume a lower-risk injury without confirmation of the final grade. Similarly, a systematic review of 484 open pilon fractures found no significant pooled difference in deep infection or nonunion between ORIF and definitive external fixation, although the evidence was limited by heterogeneous cohorts and selection bias [3]. A recent randomized trial in complex AO/OTA 43-C fractures found better ankle scores and earlier union and return to work with two-stage ORIF than with limited internal fixation plus definitive external fixation, without a detected infection difference [8]. Collectively, these findings support tailoring definitive fixation to fracture morphology and soft tissue conditions.

Patient factors also shape the interpretation of this case. Vascular status, pre-existing skin disease, medication history, and glycemic control are relevant when assessing readiness for definitive fixation. Diabetes has been associated with substantially more infection and nonunion after operative treatment of pilon fractures. In a single-center cohort, Kline et al. reported infection in 71% of fractures in patients with diabetes compared with 19% in those without diabetes, and nonunion or delayed union in 43% compared with 16%, although the diabetic group included only 14 fractures [9]. Against this background, the absence of clinical infection through eight weeks is encouraging but provisional because deep infection and nonunion after pilon fixation may become apparent later. The absence of early clinical infection cannot be attributed to the timing or any individual component of treatment in a single case.

The unfixed fibular fracture and the shortening with slight extension deformity noted at eight weeks also warrant consideration. Restoration of fibular length has traditionally been one of the steps of pilon reconstruction, but fibular fixation adds an incision, and its role remains debated [1]. In a national database study of 3,120 patients with closed pilon fractures, fibular fixation during ORIF was not associated with higher 30-day rates of surgical site infection or wound dehiscence, although it was associated with more reoperations [10]. A comparative series found no difference in final alignment between selected patients treated with and without fibular fixation, so shortening should not be considered inevitable when the fibula is left unfixed [11]. In this patient, the fibula was allowed to heal nonoperatively, so length depended on the tibial construct. Whether the radiographic shortening reflects settling through metaphyseal comminution, loss of reduction, or differences in projection cannot be determined from the available images, and its clinical significance will depend on standing radiographs and later functional follow-up. Some surgeons fix the fibula at the time of external fixation to help maintain length before definitive reconstruction; this case cannot show whether that would have changed the radiographic result.

This report has important limitations. The available clinical documentation did not provide a formal measure of glycemic control, admission laboratory values, tetanus management, a detailed initial neurovascular examination, CT findings, other injuries identified during the trauma evaluation, a final AO/OTA classification, or the specific soft tissue findings used to proceed with definitive fixation on day four. Follow-up was limited to eight weeks, precluding assessment of union, final alignment, ankle motion, weight-bearing progression, return to activity, reoperation, post-traumatic arthritis, or patient-reported function. The observed outcome is therefore limited to early wound status, neurologic examination, and early radiographic findings. This case illustrates an individualized treatment sequence but cannot establish the safety or superiority of early conversion.

## Conclusions

An open pilon fracture with an associated fibular fracture was managed with debridement, limited percutaneous fixation, temporary ankle-spanning external fixation, and definitive tibial ORIF four days later. Through eight weeks, there was no clinical evidence of infection despite the patient's diabetes, and radiographs showed callus formation with intact hardware, although some shortening and a slight extension deformity were noted. The case demonstrates that early staged conversion can be incorporated into individualized management, but it does not define an optimal interval or resolve the role of fibular fixation. Definitive timing should remain based on the open wound, soft tissue condition, fracture pattern, patient factors, and the requirements of the planned reconstruction.
